# Impact of high-altitude acclimatization and de-acclimatization on the intestinal microbiota of rats in a natural high-altitude environment

**DOI:** 10.3389/fmicb.2024.1371247

**Published:** 2024-05-07

**Authors:** Doudou Hao, Haomeng Niu, Qin Zhao, Jing Shi, Chuanhao An, Siyu Wang, Chaohua Zhou, Siyuan Chen, Yongxing Fu, Yongqun Zhang, Zeng He

**Affiliations:** ^1^Biobank, Hospital of Chengdu Office of People’s Government of Tibetan Autonomous Region, Chengdu, China; ^2^Medical College, Tibet University, Lhasa, China; ^3^Health Clinic, Training Base of the Armed Police Force of Tibet, Lhasa, China

**Keywords:** high altitude hypoxia, gut microbiota, high-altitude acclimatization, high-altitude de-acclimatization, plateau environment

## Abstract

**Introduction:**

Intestinal microorganisms play an important role in the health of both humans and animals, with their composition being influenced by changes in the host’s environment.

**Methods:**

We evaluated the longitudinal changes in the fecal microbial community of rats at different altitudes across various time points. Rats were airlifted to high altitude (3,650 m) and acclimatized for 42 days (HAC), before being by airlifted back to low altitude (500 m) and de-acclimatized for 28 days (HADA); meanwhile, the control group included rats living at low altitude (500 m; LA). We investigated changes in the gut microbiota at 12 time points during high-altitude acclimatization and de-acclimatization, employing 16S rRNA gene sequencing technology alongside physiological indices, such as weight and daily autonomous activity time.

**Results:**

A significant increase in the Chao1 index was observed on day 14 in the HAC and HADA groups compared to that in the LA group, indicating clear differences in species richness. Moreover, the principal coordinate analysis revealed that the bacterial community structures of HAC and HADA differed from those in LA. Long-term high-altitude acclimatization and de- acclimatization resulted in the reduced abundance of the probiotic Lactobacillus. Altitude and age significantly influenced intestinal microbiota composition, with changes in ambient oxygen content and atmospheric partial pressure being considered key causal factors of altitude-dependent alterations in microbiota composition. High-altitude may be linked to an increase in anaerobic bacterial abundance and a decrease in non-anaerobic bacterial abundance.

**Discussion:**

In this study, the hypobaric hypoxic conditions at high-altitude increased the abundance of anaerobes, while reducing the abundance of probiotics; these changes in bacterial community structure may, ultimately, affect host health. Overall, gaining a comprehensive understanding of the intestinal microbiota alterations during high-altitude acclimatization and de-acclimatization is essential for the development of effective prevention and treatment strategies to better protect the health of individuals traveling between high- and low-altitude areas.

## 1 Introduction

Approximately 14.4 million people globally live at altitudes ≥3,500 m, with China hosting the largest population at such elevations (Tremblay and Ainslie, 2021). Given the frequent travel between high-and low-altitude regions and the short durations spent in either environment, high-altitude acclimatization and de-acclimatization are considered continuous processes. However, poor acclimatization to high altitudes can result in pathological damage (Khanna et al., 2018), often manifesting as symptoms such as indigestion, bloating, and diarrhea in individuals with brief exposure to high altitudes (Anand et al., 2006). Previous studies have highlighted the impact of a high-altitude hypoxic environment on colonic inflammation (Luo et al., 2022), intestinal mucosal injury, atrophy, and barrier dysfunction (Zhang et al., 2015). Moreover, high-altitudes have been found to affect the expression pattern of duodenal solute carrier (SLC) transporters, thus affecting intestinal transport function (Wojtal et al., 2014). Hypobaric hypoxia–induced microbial dysbiosis can alter the expression of drug-metabolizing enzymes and transporters, thereby influencing the effectiveness and safety of drugs *in vivo* (Zhang J. et al., 2018; Bai et al., 2022). High-altitude environments are also considered risk factors for anxiety and depression (Sheth et al., 2018), accompanied by reduced activation of neural circuits for cravings, leading to decreased appetite and weight loss (Yan et al., 2011).

Environmental changes can rapidly alter the composition and function of the intestinal microbiota, with this remodeling effect surpassing the influence of host genetics (Liu et al., 2021). Homeostasis of the intestinal microbiota is maintained by the intricate balance between different bacterial communities, intestinal epithelial tissues, and the host immune system, achieved through constant adjustments of internal parameters (Wang et al., 2024). However, various factors, such as exogenous substances (e.g., antibiotics; Tong et al., 2024), sleep deprivation (Yao et al., 2022), immune imbalance, and pressure, can disrupt this homeostasis (Tanaka and Nakayama, 2017). Different altitude result in the development of distinct intestinal microbiota compositions and structures, ultimately forming unique intestinal microbiota communities that are adapted to this environment. *Bacillus subtilis* and *Bacillus velezensis*, isolated from Tibetan yaks, can enhance overall growth performance and ameliorate blood parameters related to inflammation and immunity in mice (Li et al., 2019). Moreover, *Prevella_1*, the dominant genus in Tibetan sheep, has been found to promote feed fermentation, produce high concentrations of short chain fatty acid (SCFA), minimize energy loss, and enhance adaption to high-altitude environments (Lv et al., 2021).

Bacteria can be classified according to their oxygen demand into obligate anaerobes, facultative anaerobes, aerotolerant anaerobes, microaerophiles, and obligate aerobes (Suzuki et al., 2019). The gut serves as a highly intricate and diverse microbial ecosystem, hosting a myriad of microbial species. While a microaerobic layer exists in the brush border region, the majority of intestinal tracts (Miller et al., 2020), particularly the colon, constitute stringent anaerobic environments, with anaerobic bacteria playing a predominant role (De Vos et al., 2022). Under such conditions, anaerobic bacteria may have a competitive advantage over aerobic bacteria, leading to an increased proliferation of anaerobic bacterial populations. Obligate and facultative anaerobes generate large amounts of acid and gases, which can lead to increased flatulence during high-altitude stress and, more severely, to acid accumulation and indigestion (Adak et al., 2014). In high-altitude populations, the abundance of aerobic bacteria is notably lower, with high-altitude Tibetan populations exhibiting the lowest abundance, followed by high-altitude Han populations; meanwhile, low-altitude Han populations possessed relatively high abundances of aerobic bacteria (Jia et al., 2020). Moreover, a study on soldiers undergoing training from low to high altitudes (3,500 m) revealed an increase in the total number of obligate and facultative anaerobes, alongside a decrease in the total number of fecal aerobic bacteria (Adak et al., 2013). Additionally, results from an intestinal intervention therapy study suggest that the intestinal microbiota may serve as a pathogenic factor for high-altitude-associated morbidity, presenting a promising strategy for the treatment of high-altitude populations (Hu et al., 2022). Irbesartan has been found to reduce oxidative damage–induced altitude hypoxia and pulmonary edema in rats; this effect has been attributed to the increased abundance of *Lactobacillaceae* and *Lachnospiraceae* and the decreased abundance of *Prevotellaceae* and *Desulfovibrionaceae* in the intestines of these irbesartan-treated rats (Nijiati et al., 2021).

When individuals return from high to low altitudes, they undergo a period of high-altitude de-acclimatization, with the duration of this process varying among individuals. Reoxygenation injury during de-acclimatization may lead to an imbalance in inflammatory factors, which may induce inflammatory reactions, morphological changes in the carotid body, pH alterations in the cerebrospinal fluid, and physiological changes in the central nervous system (Woolley et al., 1963; Dempsey et al., 1979; Matsuda et al., 2006). Following reoxygenation injury, the increase in erythrocyte volume and polycythemia has been found to persist for 1 month, with cardiorespiratory changes disappearing after 1 month and complete recovery being observed after 2 months upon returning to a low-altitude environment (Savourey et al., 1996). A cohort study demonstrated that approximately 100 days were needed for blood cell levels to return to baseline in individuals undergoing high-altitude de-acclimatization (He et al., 2013).

Altitude, transportation processes, environmental humidity, atmospheric pressure, and oxygen content collectively affect the development of the intestinal microbiota in both humans and animals, influencing interactions between of microbes. The rapid changes in atmospheric pressure and oxygen content experienced during travel from low-elevation plains to high-elevation plateaus, followed by a return to the plains, can lead to alterations in the intestinal microbiota of animals and humans. However, the specific changes in the intestinal microecology and its association with altitude-related illnesses remains unclear. Currently, there is a lack of continuous monitoring studies on the gut microbiome during high-altitude acclimation and de-acclimation in natural high-altitude environments. Therefore, developing a greater understanding of elevation-related alterations in the gut microbiota is important. In this study, we aimed to provide a reference for understanding the changes in the intestinal microbiota of individuals traveling between low and high altitude, offering valuable insights that can enhance public health guidance.

## 2 Methods

### 2.1 Experimental design

**Figure 1:**
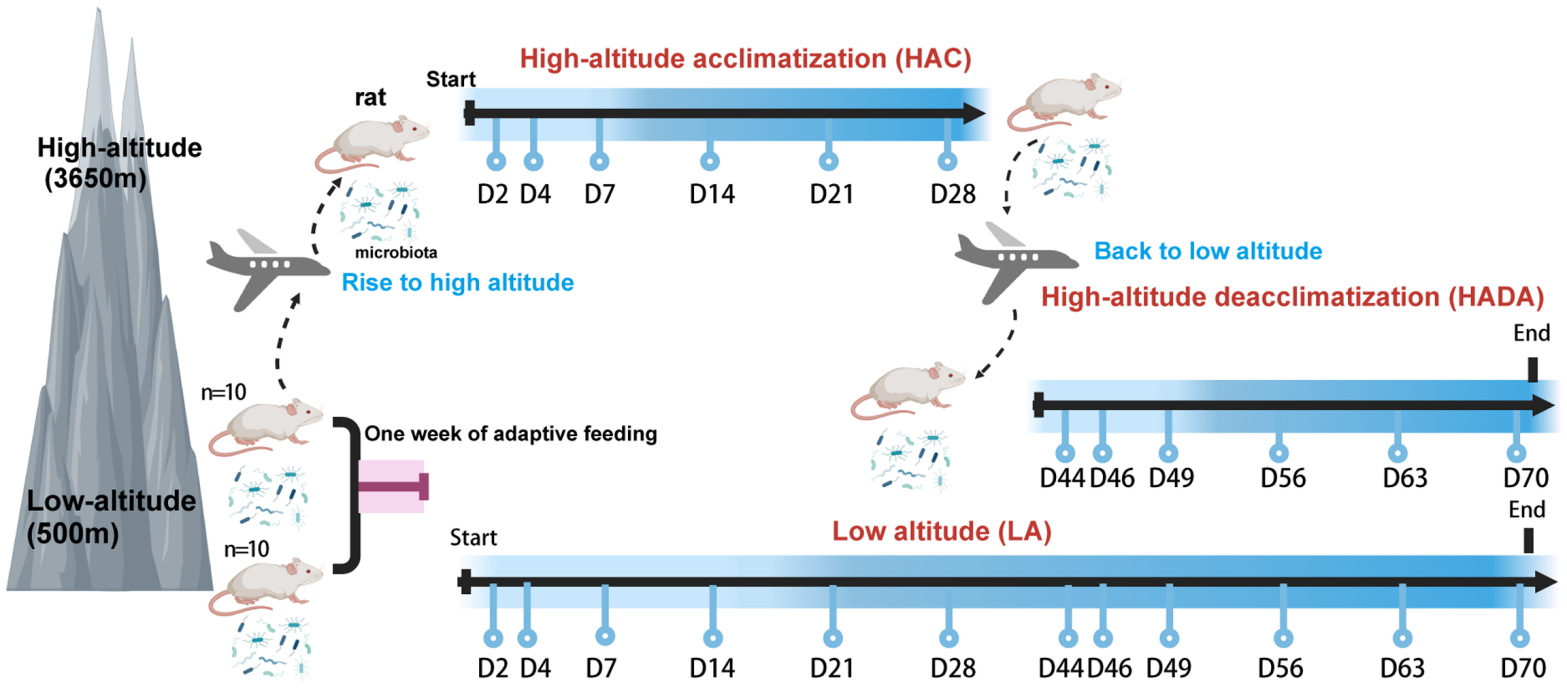
Experimental design of this study. Created with BioRender.com.

In this study, we investigated the dynamic changes in the gut microbiota of rats during high-altitude acclimatization and de-acclimatization. In total, 20 rats were divided into two groups. The control group (*n* = 10) was maintained under normoxic conditions at low altitude (LA) (Chengdu, China; 500 m). The experimental group (*n* = 10) was further subdivided into two distinct research stages, resulting in two distinct subsets within the experimental group. The first stage involved high-altitude acclimatization (HAC), whereby rats were transported by airplane from low altitude (Chengdu, China; 500 m) to high altitude (Lhasa, China; 3,650 m) and monitored for high-altitude acclimatization for 2, 4, 7, 14, 21, and 28 d. The second stage involved high-altitude de-acclimatization (HADA), whereby the rats were transported by airplane from high altitude (Lhasa, China; 3,650 m) to low altitude (Chengdu, China; 500 m) and monitored for high-altitude de-acclimatization for 2, 4, 7, 14, 21, and 28 d. An adaptive feeding period of 1 week was conducted prior to the experiments. The experimental design and timeline of this study are shown in Figure 1. Stool samples were collected at 12 different time points using sterile 2 mL centrifuge tubes, rapidly transferred to liquid nitrogen for quick freezing, then transported on dry ice, and stored at-80°C in biobank for later analysis. Subsequently, the intestinal microbiota in the stool samples (*n* = 240) were subjected to 16S rRNA sequencing. This study was approved by the Ethics Committee of Hospital of Chengdu Office of People’s Government of Tibetan Autonomous Region, China [Scientific Research No. 10 (2023)].

### 2.2 Animal experiments

Healthy 9–10 week-old male Sprague Dawley rats (*n* = 20), weighing approximately 220 ± 10 g, were purchased from Chengdu Dossy Biological Technology [Chengdu, China; certificate number: SCXK (Chuan) 2022–030]. The rats were housed in a rat box (460 × 315 × 200 mm) with one rat per cage. During high-altitude acclimatization, the rats were housed in the Animal Laboratory of the General Hospital of the Tibet Military Region of the Chinese People’s Liberation Army. The rats were maintained under a natural light cycle at high-altitude areas at a temperature of 18 ± 4°C; the humidity in Lhasa is 10–40%, while that in Chengdu is 30–60%. The rats were allowed *ad libitum* access to water and food. Daily autonomous activity time was monitored using an autonomous activity recorder (Shanghai Yuyan Scientific Instrument; YLS-1C) at each sampling point. The automatic activity recorder detected the change and displacement of infrared energy radiated by the rats without direct contact; the recorder then converted the changing parameters into voltage signals to provide automatic monitoring, with activity time being calculated in seconds. Body weight was monitored at each sampling point. The current research has consistently adhered to ethical principles and strives to minimize the impact on animal welfare. In determining the sample size for the study, we comprehensively reviewed previous research on the gut microbiota of rats and conducted a thorough analysis of the sample sizes used in these studies (Liao et al., 2023; Wang et al., 2023). This allowed us to establish the range of animal numbers required to obtain reliable statistical results. Given that previous research has revealed consistency in gut microbiota patterns, we ultimately decided to use 10 rats per group for the experiments. This decision aims to ensure the scientific rigor and precision of the study while minimizing the use of experimental animals.

### 2.3 Genomic DNA extraction and 16S rRNA gene sequencing

The genomic DNA was extracted using cetyltrimethylammonium bromide. Then, PCR amplification of the variable region V3 + V4 was conducted using 341F (5′-CCTAYGGGRBGCASCAG-3′) and 806R (5′-GGACTACNNGGGTATCTAAT-3′) primers. A corresponding sequencing library was generated using the Truseq DNA PCR-Free Sample Preparation Kit (Illumina, United States). Subsequently the DNA library was sequenced on an Illumina NovaSeq PE250 platform, yielding 250 bp pair-end sequences.

### 2.4 Gut microbiota analysis based on 16S rRNA gene sequencing

Gut microbiota analysis was conducted according to procedures outlined in the Qiime2 document “Atacama Soil Microbiology Course”.^1^ All 16S rRNA datasets were analyzed using the DATA2 plugin of Qiime2, enabling filtering, denoising, merging, non-chimerism, and amplicon sequence variant (ASV) generation. ASVs were assigned based on the Greengenes Database (version 13.8) to obtain species annotation information. To estimate the microbial diversity within individual samples, we calculated feature-level alpha diversity indices such as the Chao1 richness estimator, Shannon diversity index, and Simpson diversity index. To gain insights into the beta diversity among our samples, we conducted distance measurements using the Bray–Curtis dissimilarity index, which was subsequently visualized through principal coordinate analysis (PCoA) for a comprehensive understanding of the patterns and relationships among the microbial communities (Vázquez-Baeza et al., 2013). To identify differences in the relative abundance of species among populations, we utilized the Statistical Analysis of Taxonomic and Functional Profiles (STAMP) software, employing Welch’s t-test. Moreover, we conducted Spearman analysis to investigate relationships between microbial communities, weights, and daily activity times based on the relative abundance of microbial species at various taxonomic levels. This analysis provided insights into the potential ecological and functional roles of microbial species. Additionally, we employed the LEfSe analysis method (Segata et al., 2011) to perform a comprehensive comparison between two datasets. Initially, we used the non-parametric factorial Kruskal-Wallis and rank test (KW test) to identify species with significant abundance differences among groups. Subsequently, we validated these differences using the grouped Wilcoxon rank sum test. Finally, linear discriminant analysis (LDA) was conducted for dimension reduction and to assess the impact of species with significant differences, generating LDA scores. We set the LDA threshold to be greater than 4 to ensure statistical significance. To predict the potential functional distribution of the intestinal microbiota, we utilized PICRUSt software (Langille et al., 2013). Finally, the microbiome phenotypes of the bacteria were classified using BugBase software, providing a comprehensive understanding of the microbial composition and its functional implications (Ward et al., 2017).

### 2.5 Statistical analysis

Descriptive results are presented via mean ± standard deviation for continuous variables. Normality of the data distribution was assessed based on the Shapiro–Wilk test. The Levene’s test was utilized to evaluate the homoscedasticity of all data. The time course of statistical changes in body weight and daily autonomous activity time between the two groups was evaluated using one-way analysis of variance (ANOVA) for repeated measures. Following the ANOVA analysis, to determine more precisely which groups or conditions exhibit significant differences, we conduct follow-up tests and adjust the significance level using Bonferroni correction to control the probability of Type I error. When significant interactions are detected, we adopt the Benjamini-Hochberg method to further adjust the significance level and control the false discovery rate in multiple comparisons. To examine the significance of differences in alpha diversity indices, including Chao1, Shannon, and Simpson, the Wilcoxon rank-sum test was utilized. For the study of beta diversity, a non-parametric multivariate analysis of variance based on Bray-Curtis’s distance was employed to measure species composition differences. This approach enabled us to assess the variability and differences in microbial composition across samples.

Repeated measures ANOVA was performed to evaluate statistical significance using GraphPad Prism Version 8. *p* < 0.05 was considered statistically significant.

### 2.6 Data availability

Raw reads from amplicon sequencing were deposited in the NCBI Sequence Read Archive database (BioProject numbers: PRJNA910368, PRJNA909981, and PRJNA740353).

## 3 Results

### 3.1 Altitude-associated changes in body weight and daily autonomous activity time

**Figure 2:**
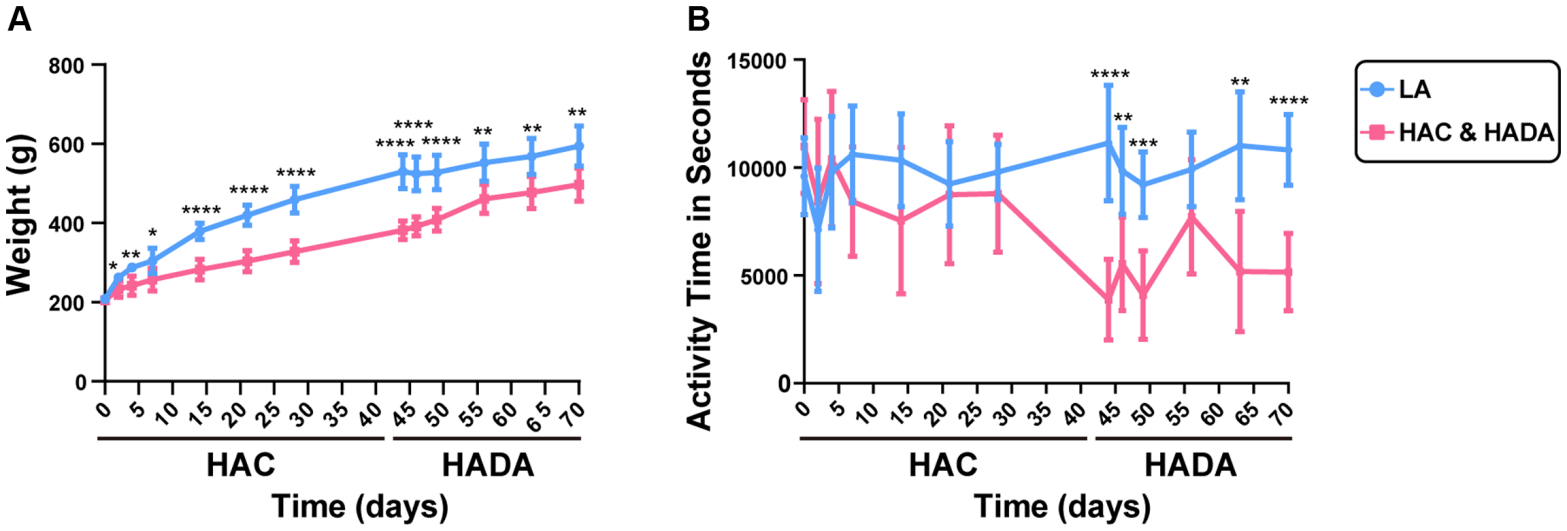
**(A)** Changes in the body weight of rats during high-altitude acclimatization (HAC) and de-acclimation (HADA). **(B)** Comparison of the autonomic activity time (s) of rats during HAC and HADA with the low altitude (LA) group. *p < 0.05; **p < 0.01; ***p < 0.001; ****p < 0.0001.

In this study, we observed a significantly lower increase in the rate of body weight gain in HAC rats than those in the LA group; this difference in weight gain became significant on day 14. By contrast, during HADA, the gain in body weight significantly increased on day 14 (Figure 2A). No significant difference in the autonomous activity time was observed in HAC rats compared to that in the control group; however, daily autonomous activity time was significantly reduced in HADA rats (Figure 2B).

### 3.2 High-altitude acclimatization and HADA induce a significant increase in the abundance of microbiota on day 14

**Figure 3:**
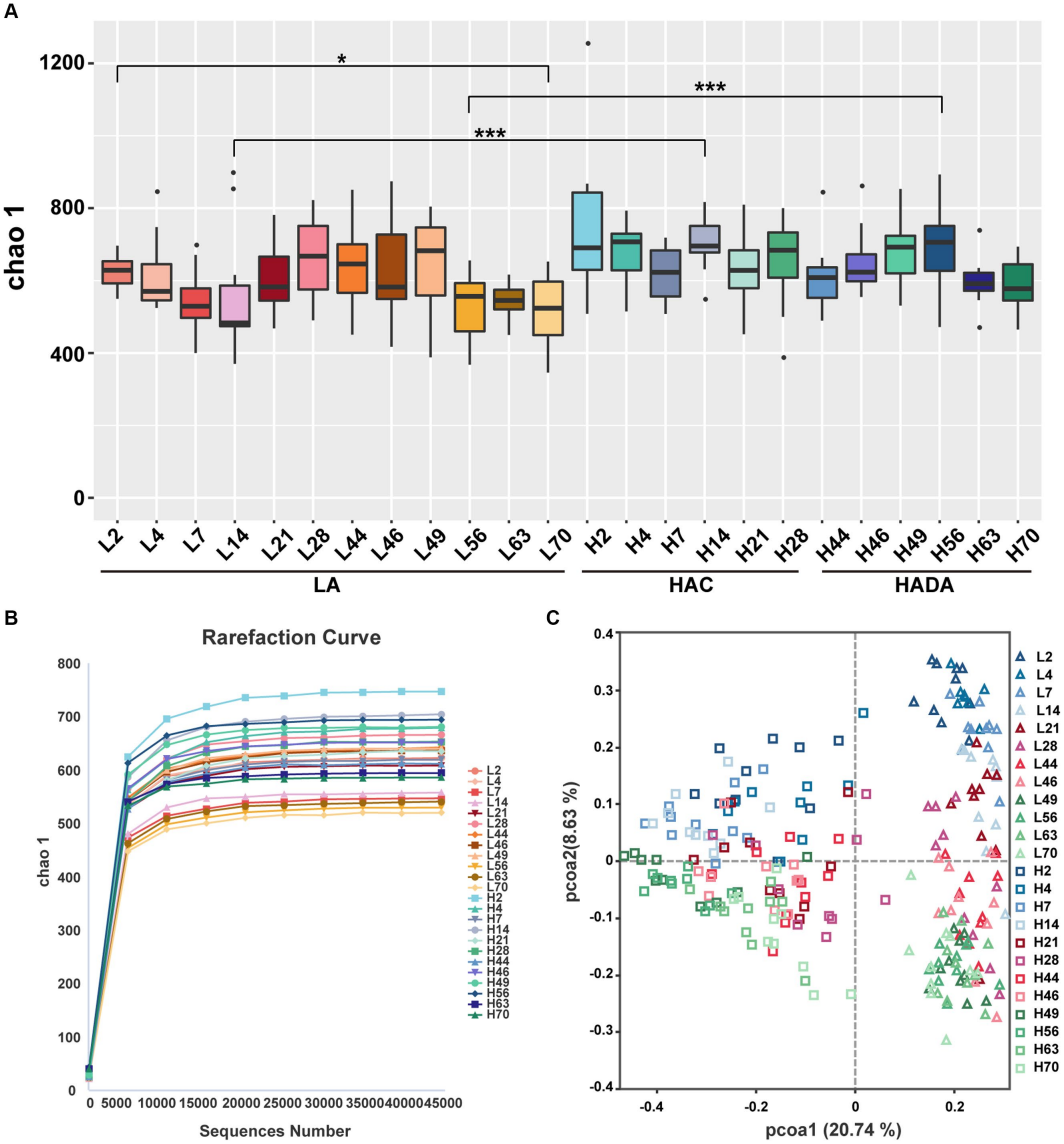
Alpha and beta diversity analysis. **(A)** Statistical analysis of Alpha diversity index Chao1. Wilcoxon rank-sum test was used to examine the significance of differences in alpha diversity indices chao1between two groups. LA, low altitude; HAC, high-altitude acclimatization; HADA, high-altitude de-acclimatization. The X-axis denotes different time points. *p < 0.05; ***p < 0.001. **(B)** Dilution curves produced at different time points in the different groups. **(C)** Analysis of PCoA based on Bray–Curtis dissimilarity. Triangles represent the low altitude (LA) group, while squares represent the high altitude acclimatization (HAC) and high altitude de-acclimatization (HADA) groups. The 2nd-14th day, the 21st-46th day, and the 49th-70th day are marked with blue, red, and green gradient colors, respectively.

A similar trend was observed in the microbial microbiota during both HAC and HADA. On day 14, the Chao1 index of the HAC and HADA groups was found to be significantly higher than that of the LA group, indicating an increase in the total number of species in these experimental groups, particularly low-abundance species (Figure 3A). These findings suggest that changes in the altitude environment may lead to an increase in low-abundance species, with the most significant difference being observed on day 14. Dilution curve analysis confirmed that the sample size was sufficient and the alpha diversity index was stable in all groups (Figure 3B). The two other alpha diversity indices (Shannon and Simpson index) indicated no significant differences between the groups (Supplementary Figure S1). Subsequently, PCoA analysis revealed the sample distribution, with different shapes representing the low-altitude control (LA) and experimental groups (HAC and HADA), and different colors indicating various time points. Overall, the HAC, HADA, and LA groups formed two distinct clusters; in this analysis further distribution distances indicated greater differences in the population structure of the microbial microbiota (Figure 3C).

### 3.3 Relative abundance of intestinal microbiota at different time points during HAC and HADA

**Figure 4:**
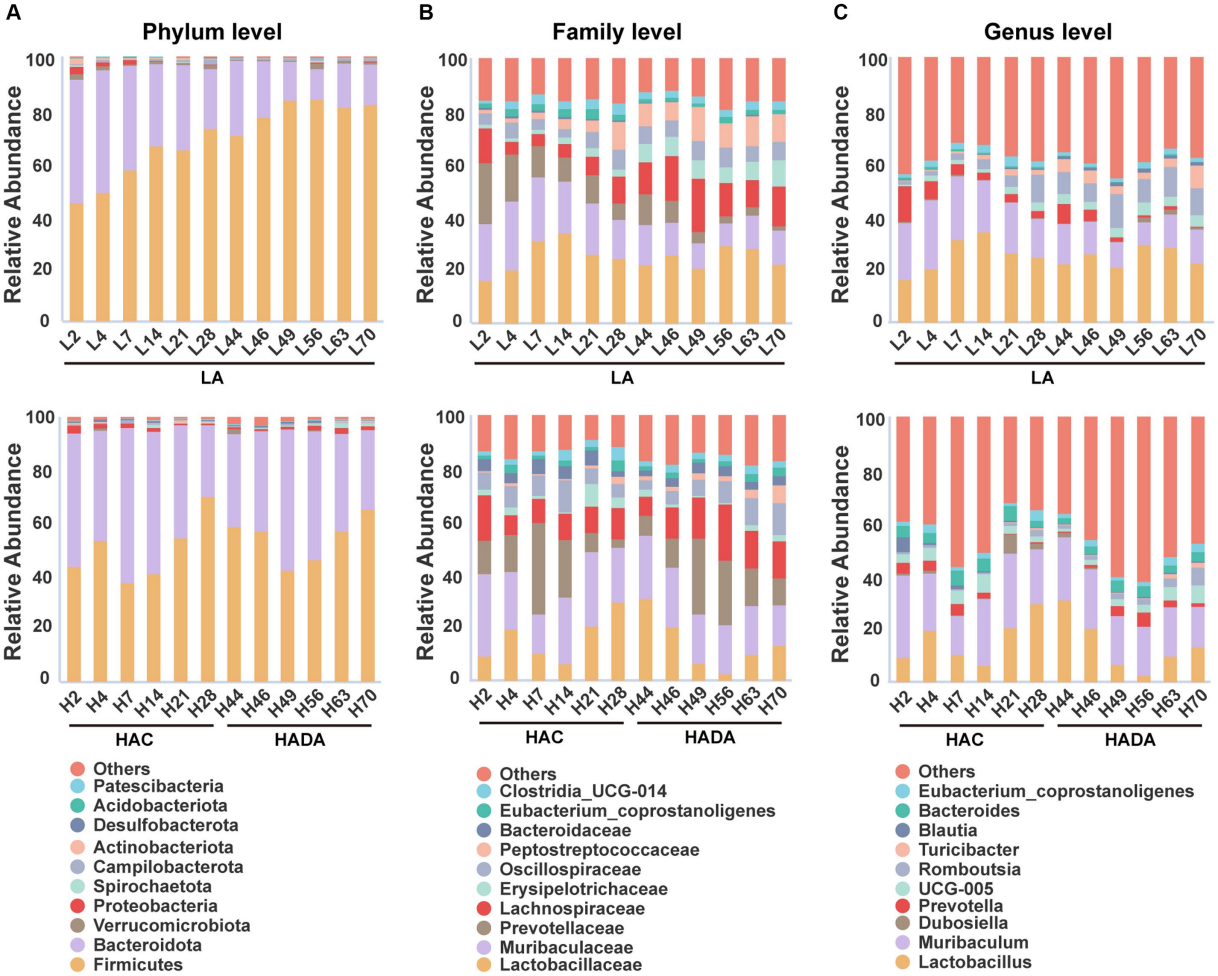
**(A-C)** The relative abundances among the three groups at the phylum, family, and genus levels. LA, low altitude; HAC, high-altitude acclimatization; HADA, high-altitude de-acclimatization. The X-axis denotes different time points in different groups.

In this study, we revealed that Firmicutes and Bacteroidota were the predominant phyla in the gut microbiome of rats (Figure 4A). At the family level, Lactobacillaceae, Muribaculaceae, and Prevotellaceae emerged as the dominant taxa in these rats (Figure 4B). Finally, *Lactobacillus* and *Muribaculum* were identified as the most abundant bacterial genera (Figure 4C). During HAC and HADA, the abundance variation of many microbial species followed a similar trend to that observed in the LA group. In all groups, the relative abundance of Firmicutes was the lowest on day 7, gradually increasing thereafter; by contrast, the relative abundance of Bacteroidota peaked on day 7 before gradually decreasing. At the family level, the relative abundance of Lactobacillaceae initially decreased and then increased, while the relative abundance of Prevotellaceae varied greatly, exhibiting peak abundance on day 7 before gradually decreasing. At the genus level, the relative abundances of *Lactobacillus* and *Muribaculum* was the lowest on day 14, gradually increasing thereafter. Nonetheless, specific bacterial populations displayed distinct trends during HAC and HADA. For example, during HAC, the relative abundance of *Blautia* spp. peaked on day 2 before rapidly reducing thereafter; meanwhile, the relative abundance of *Prevotella* spp. decreased throughout. By contrast, during HADA, the relative abundance of *Prevotella* initially increased to a peak on day 14, before decreasing.

In the LA control group, the aging of rats also altered the abundance of different intestinal microbiota over the 70-day experimental period. Notably, the relative abundance of Firmicutes gradually increased, while that of Bacteroidota gradually decreased. Moreover, at the family level, the relative abundance of Muribaculaceae and Prevotellaceae showed decreasing trends, while that of Erysipelotrichaceae and Peptostreptococcaceae showed increasing trends. Finally, at the genus level, the relative abundance of *Muribaculum* and *Prevotella* decreased, whereas that of *Romboutsia* and *Turicibacter* increased.

### 3.4 Differential abundance analysis of intestinal microbiota composition

**Figure 5:**
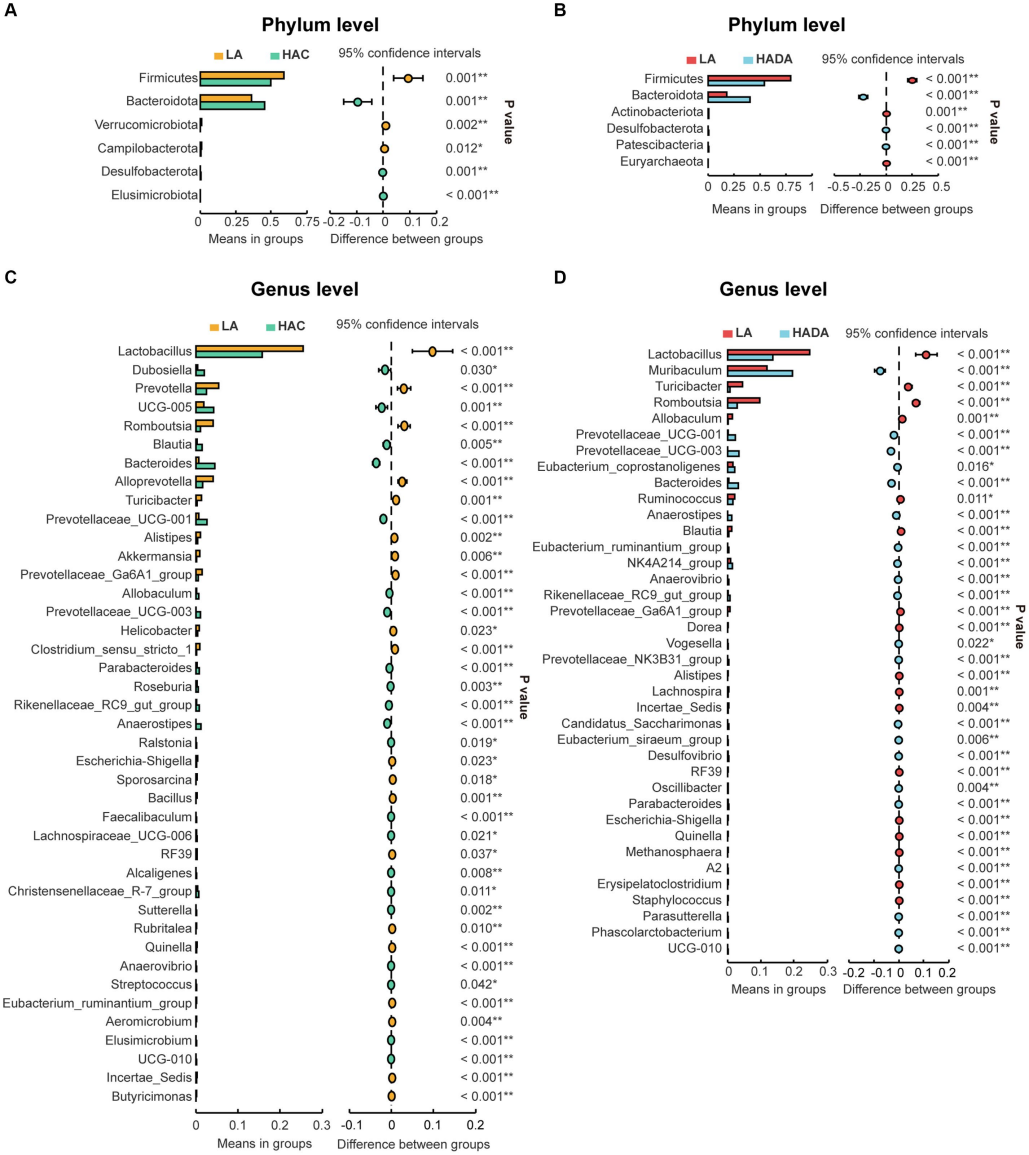
**(A, C)** Comparative analysis of gut microbiota composition at the phylum and genus levels between low altitude (LA) and high-altitude acclimatization (HAC). **(B, D)** Comparative analysis of gut microbiota composition at the phylum and genus levels between low altitude (LA) and de-acclimatization (HADA). These differences were statistically evaluated using Welch’s T-test and are presented as the corrected *P* value.

Statistical analysis was conducted to assess the differences in the relative abundance of intestinal microbiota during HAC and HADA compared with that of the LA controls. A consistent shift in specific microbiota was observed in both HAC and HADA rats. At the phylum level, the relative abundance of Firmicutes significantly decreased, whereas the relative abundance of Bacteroidota significantly increased (Figures 5A,C). At the genus level, the relative abundances of *Prevotellaceae UCG-001*, *Prevotellaceae UCG-003*, *Bacteroides*, and *Anaerostipes* increased, while the abundance of *Lactobacillus* spp. significantly decreased (Figures 5B,D). Moreover, the relative abundance of *Blautia* spp. was found to significantly increase during HAC and significantly decrease during HADA as compared to that in the LA control. Similarly, the abundance of *Prevotella* and *Akkermansia* spp. significantly decreased in the HAC group; however, no significant differences in the abundance of *Prevotella* and *Akkermansia* spp. were observed between the HADA and LA groups.

### 3.5 Identification of key microbiota and correlation analysis

**Figure 6:**
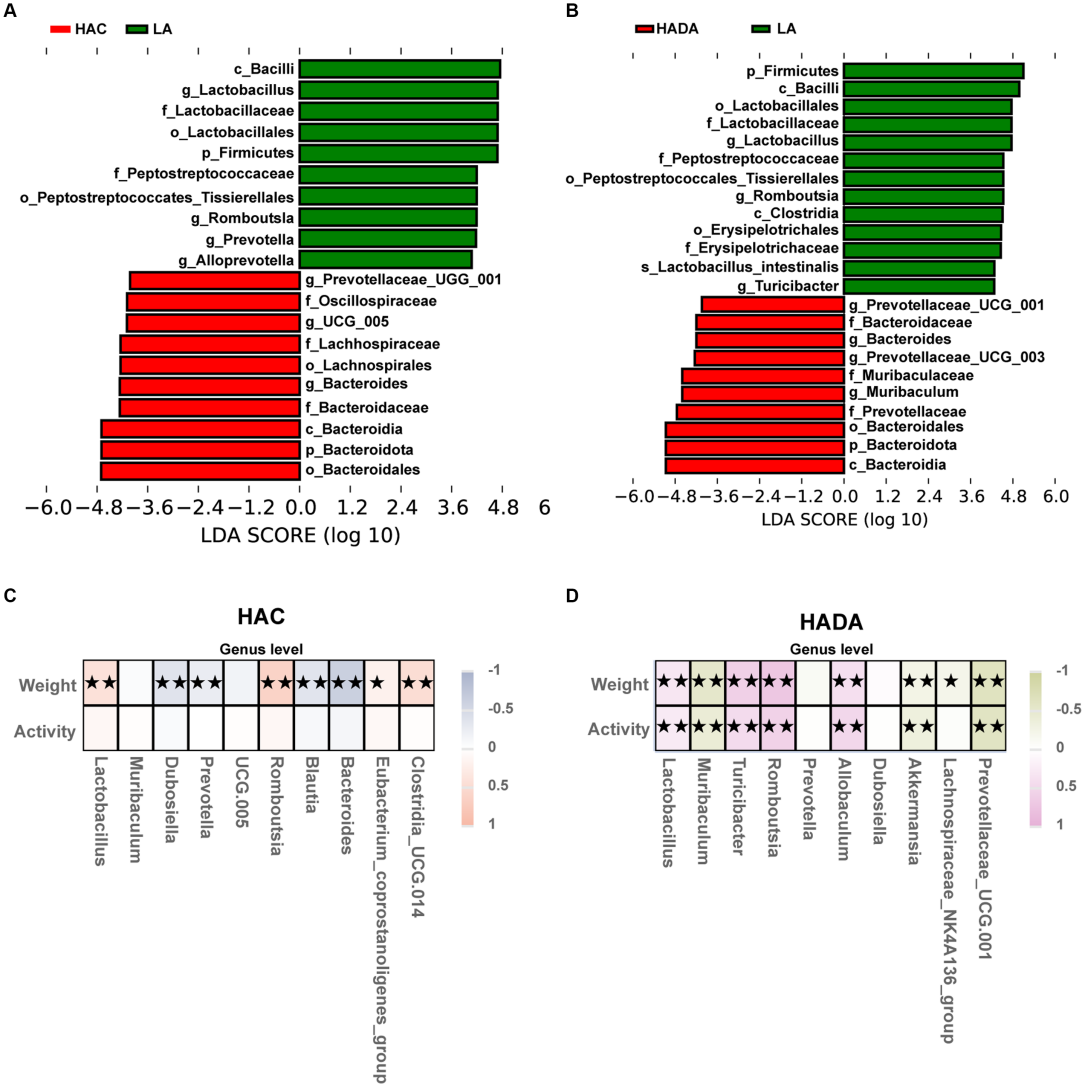
**(A,B)** Species with statistically significant differences identified through LEfSe analysis. LDA threshold was set to be greater than 4, *p* < 0.05 was considered statistically significant. LA, low altitude; HAC, high-altitude acclimatization; HADA, high-altitude de-acclimatization. **(C,D)** To assess the correlation between body weight, daily autonomous activity time, and intestinal microbiota of rats in both the HAC and HADA groups, Spearman’s test was employed. Multiple hypothesis testing correction was performed on the *p*-values using the FDR error control method (Benjamini and Hochberg False Discovery Rate). The corrected *p*-value, henceforth referred to as pFDR, was used as the criterion for statistical significance, with a threshold of pFDR <0.05. ★*p* FDR <0.05; ★★*p* FDR <0.01.

To distinguish the key microbiota with increased relative abundance in the HAC, HADA, and LA groups we employed LEfSe analysis. The LEfSe method is based on a relative abundance table that combines the Wilcoxon test (*p* < 0.05) and LDA (LDA > 4). In this analysis, a higher LDA value indicated stronger significant differences. At the genus level, *Bacteroides* was identified as the predominant microbiota in the HAC and HADA groups (Figures 6A,B).

Subsequently, correlations between body weight, daily autonomous activity time, and the intestinal microbiota of rats at the genus level were explored using Spearman’s rank correlation coefficient in HAC (Figure 6C) and HADA group (Figure 6D). Overall, *Lactobacillus* abundance was found to be positively correlated with body weight and activity time, whereas *Akkermansia* was negatively correlated with both body weight and activity time.

### 3.6 Intestinal microbial function analysis

**Figure 7:**
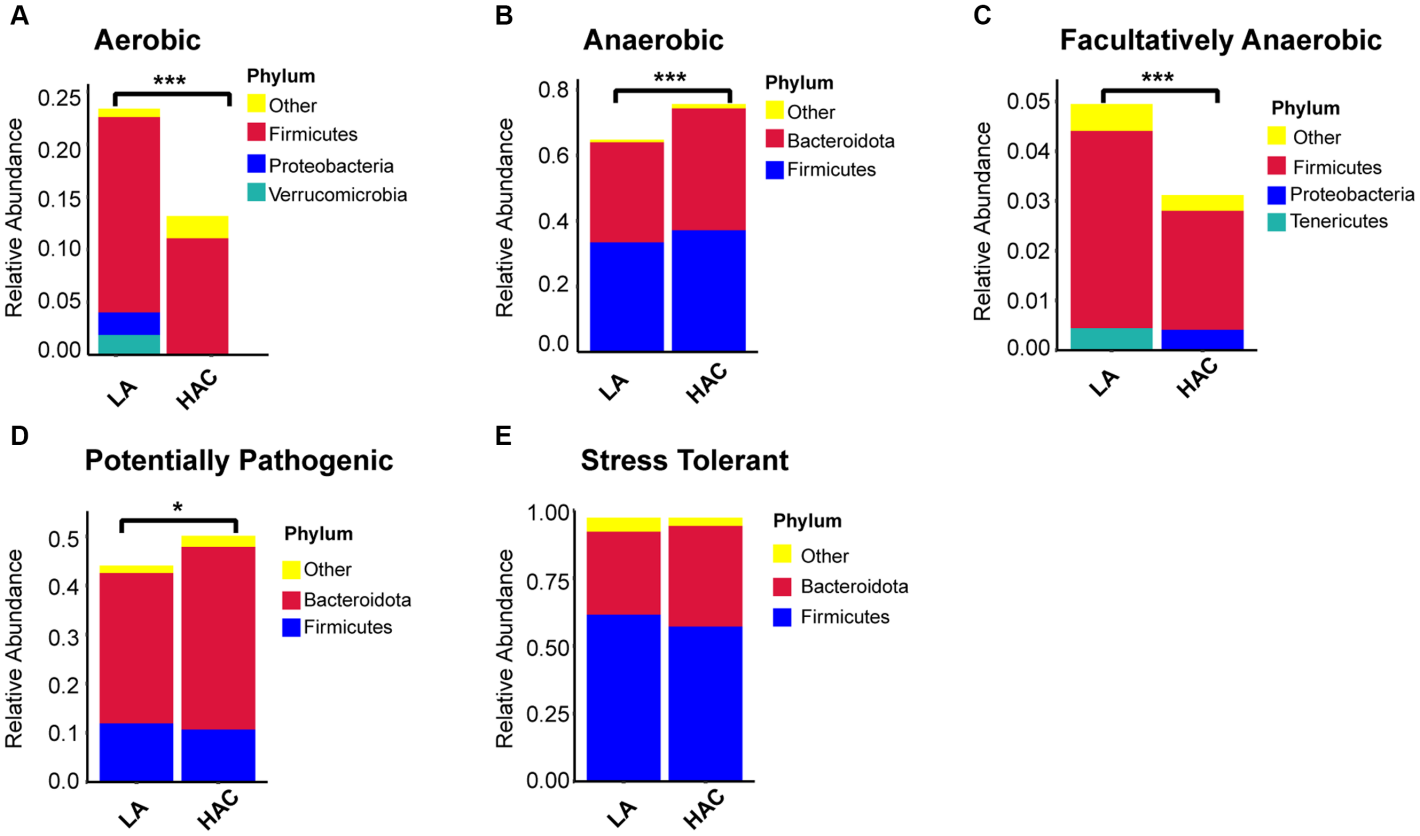
**(A-E)** Comparative analysis of the gut microbiota phenotypes during high-altitude acclimatization utilizing the BugBase database. Wilcoxon rank sum test was used to detect significant differences. LA, low altitude; HAC, high altitude acclimatization. *p < 0.05; ***p < 0.001.

**Figure 8:**
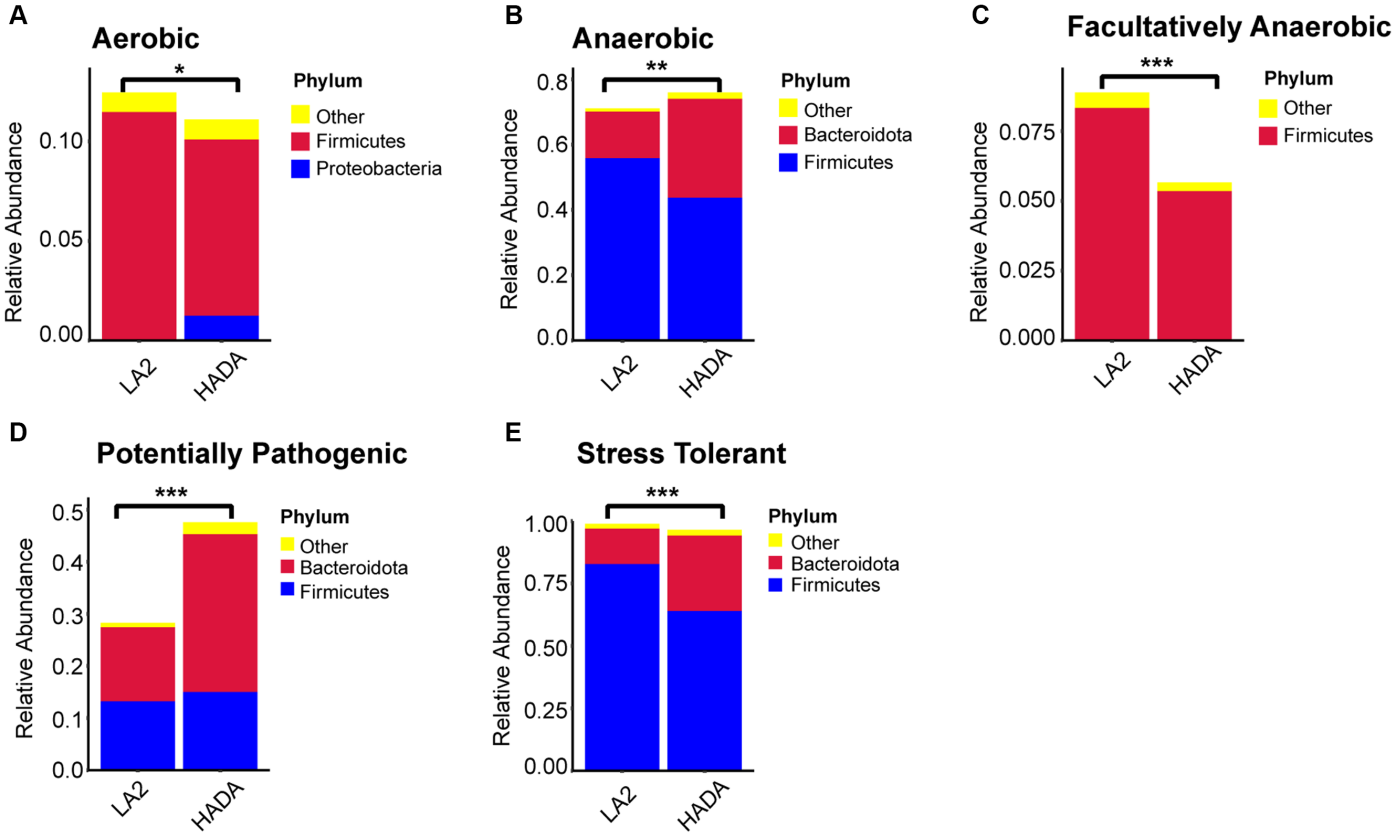
**(A-E)** Comparative analysis of the gut microbiota phenotypes during high-altitude de-acclimatization utilizing the BugBase database. Wilcoxon rank sum test was used to detect significant differences. LA, low altitude; HADA, high altitude de-acclimatization. *p < 0.05; **p < 0.01; ***p < 0.001.

**Figure 9:**
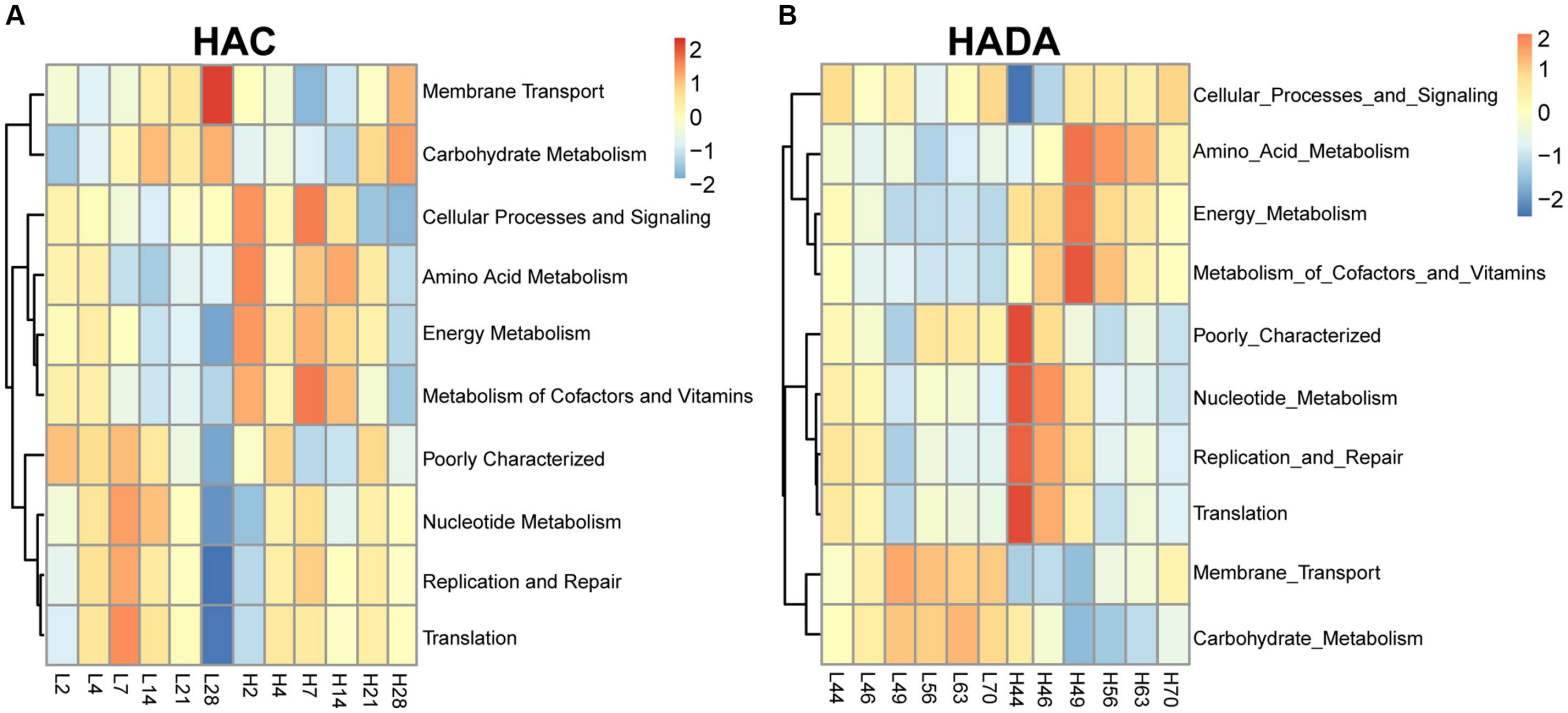
Function of the gut microbiota based on the Kyoto Encyclopedia of Genes and Genomes (KEGG) database. **(A)** H2, H4, H7, H14, H21, and H28 denote the process of high-altitude acclimatization (HAC) on day 2, 4, 7, 14, 21, and 28. L2, L4, L7, L14, L21, and L28 denote the low altitude (LA) group on day 2, 4, 7, 14, 21, and 28. **(B)** H44, H46, H49, H56, H63, and H70 denote the process of high-altitude de-acclimatization (HADA) on day 2, 4, 7, 14, 21, and 28. L44, L46, L49, L56, L63, and L70 denote the low altitude (LA) group on day 44, 46, 49, 56, 63, and 70.

Comparative analysis with the LA group revealed similar trends in the changes in microbiota function in the HAC and HADA groups (Figures 7, 8). These trends included a significant decrease in the abundance of aerobic and facultative anaerobic bacteria in the Firmicutes phylum, coupled with a significant increase in the abundance of obligate anaerobic bacteria and potential pathogenic bacteria in the Bacteroidota phylum. Interestingly, in the HAC group, the abundance of aerobic bacteria belonging to the Proteobacteria phylum decreased, while that of facultative anaerobic bacteria in the same phylum increased. Conversely, an opposite trend was observed in the HADA group: an increase in aerobic bacteria belonging to Proteobacteria and a decrease in facultative anaerobic bacteria. Furthermore, the abundance of anaerobic bacteria belonging to the Firmicutes phylum increased in the HAC group but decreased in the HADA group. Specifically, the abundance of stress-tolerant microbiota belonging to the Firmicutes phylum decreased in the HADA group. Nonetheless, in both HAC and HADA groups, the abundance of microbiota related to energy metabolism increased, whereas those related to carbohydrate metabolism decreased (Figure 9). Tolerance, in the context of microbes, generally refers to the ability of a microorganism to survive or maintain its functionality in the presence of environmental stressors. These stressors can be physical (e.g., oxygen content, temperature, pressure), chemical (e.g., toxins, solvents), or biological (e.g., predation, immune response to pathogens, sexual competition) (Sherwin et al., 2019). Hypoxia (insufficient oxygen supply) in high-altitude regions poses a significant stress to rats. It directly affects the cardiovascular system, leading to increases in heart rate and blood pressure, subsequently altering the blood supply to the intestine. This results in changes in intestinal motility, nutrient absorption, and immune function, along with elevated levels of oxidative stress, disrupting the microbial balance and thus affecting the gut microbiota. Therefore, the stress on the gut microbiota of rats in a high-altitude environment may be multifaceted, involving physiological changes in the host, altered growth conditions for microbial species, and competitive interactions within the microbiota itself.

### 3.7 Age-associated changes in intestinal microbiota

**Figure 10:**
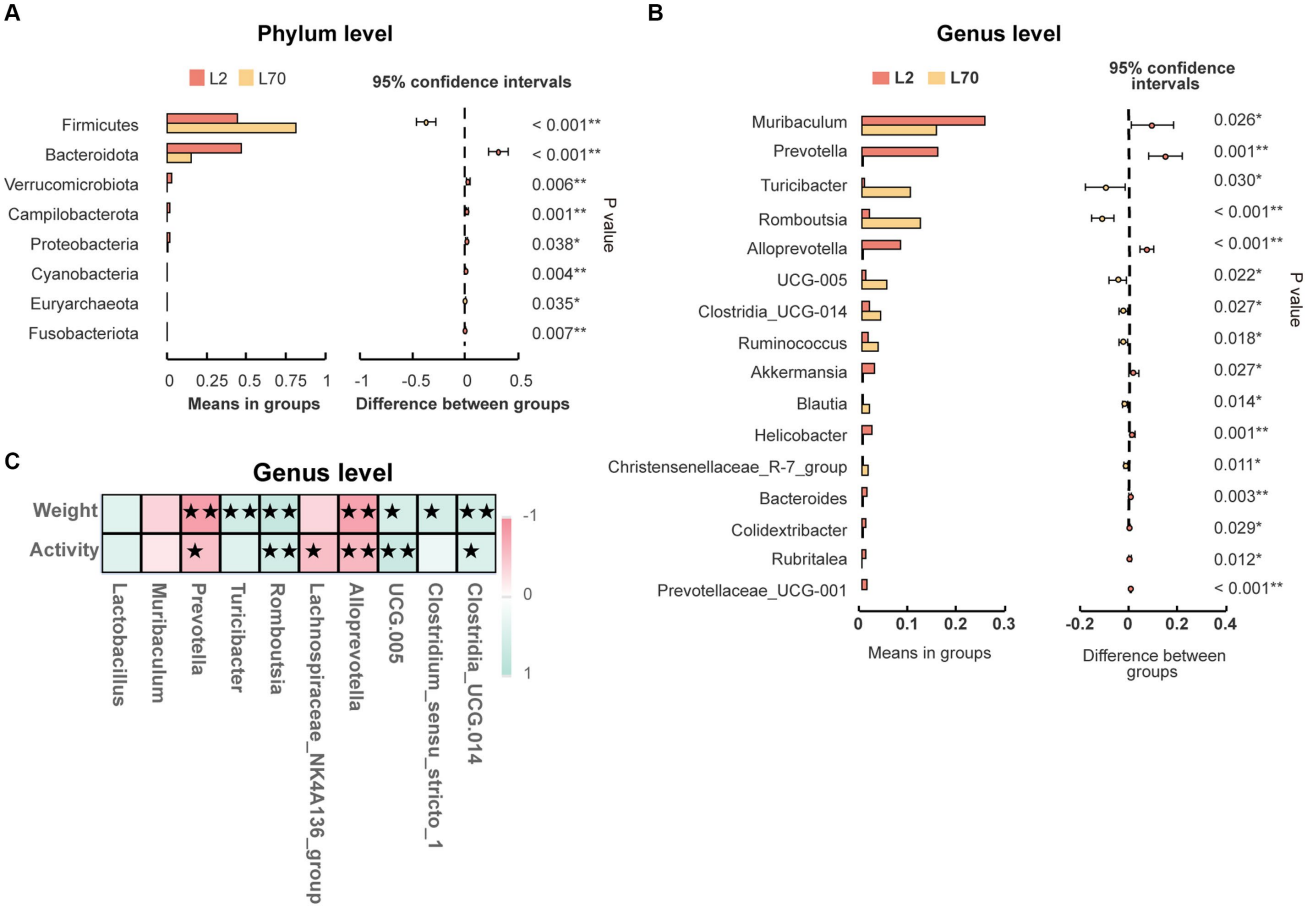
**(A,B)** Microbiota composition of day 2 (L2D) and day 70 (L70D) of the experiment, at the phylum and genus level. These differences were evaluated using Welch’s T test and expressed as the corrected *p* value. **(C)** To assess the correlation between body weight, daily autonomous activity time, and intestinal microbiota of rats, Spearman’s test was employed. Multiple hypothesis testing correction was performed on the *p* values using the FDR error control method (Benjamini and Hochberg False Discovery Rate). The corrected *p*-value, henceforth referred to as pFDR, was used as the criterion for statistical significance, with a threshold of *p*FDR <0.05. ★pFDR <0.05; ★★*p* FDR <0.01.

To demonstrate that age is indeed a key factor in intestinal microbiota changes, this study emphasizes the importance of including a low-altitude control group to more clearly reveal the unique impact of altitude changes on intestinal microbiota. Age-related changes in the intestinal microbiota were observed between day 2 (L2D) and day 70 (L70D) of the experiment, corresponding to 9–10 and 19–20 weeks old, respectively. Over these 10 weeks, the intestinal microbiota of rats changed significantly. Overall, gut microbial alpha diversity significantly decreased (*p* < 0.05) with age (Figure 3A). At the phylum level, a significant increase in the abundance of Firmicutes and a significant decrease in the abundance of Bacteroidota was observed in rats at L70D compared to those at L2D (Figure 10A). Moreover, a significant decrease in the abundance of *Muribaculum*, *Prevotella*, *Alloprevotella*, and *Akkermansia* was identified at L70D; in contrast, a significant increase in the abundance of *Turicibacter*, *Romboutsia*, and *Ruminococcus* was observed at L70D compared to that at L2D (Figure 10B). Moreover, *Prevotella* exhibited a negative correlation with body weight and daily autonomous activity time, whereas *Lactobacillus* showed a positive correlation with these factors (Figure 10C).

**Figure 11:**
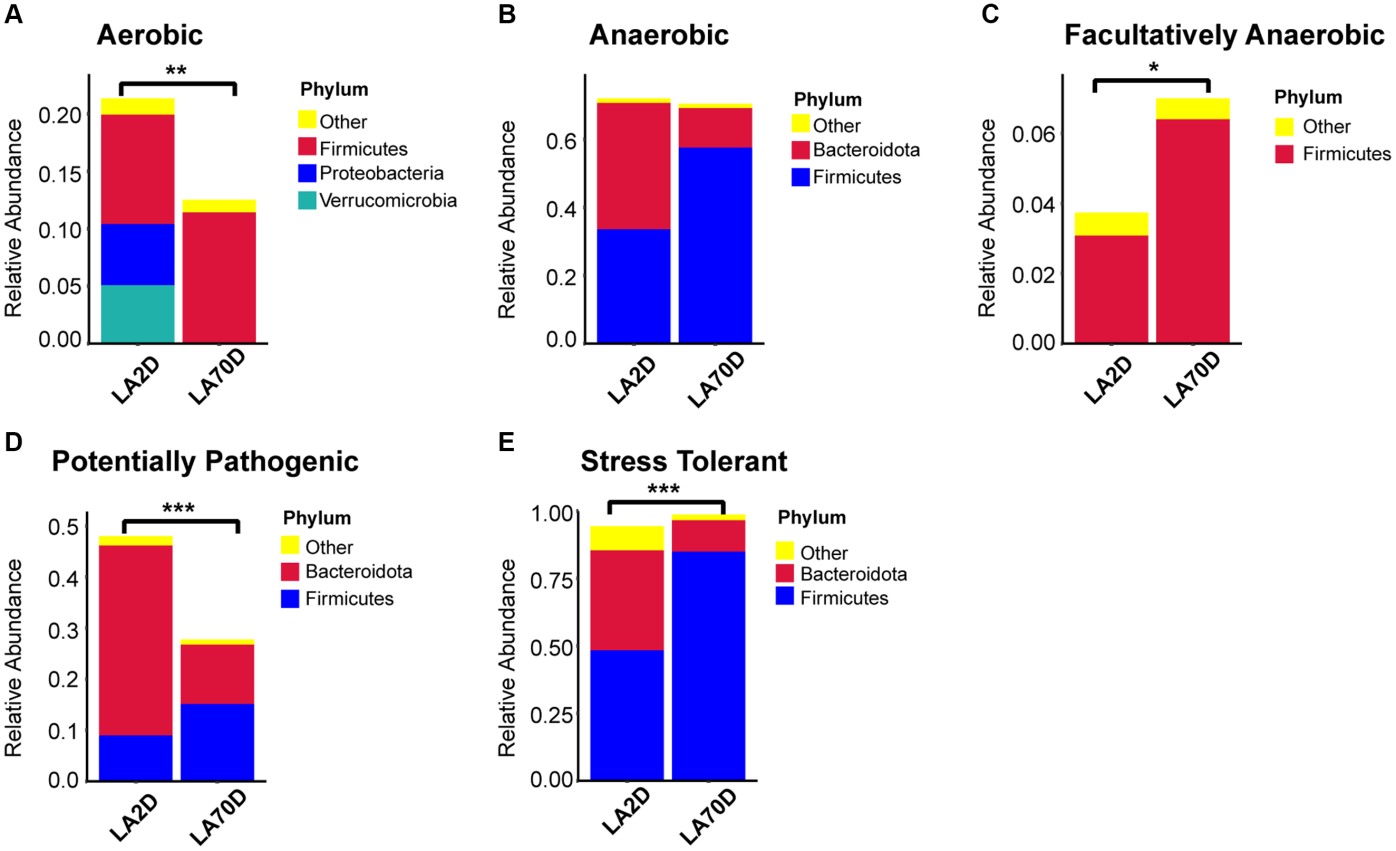
**(A-E)** Comparative analysis of intestinal microbiota function using the BugBase database. Samples were taken on day 2 (L2D) and day 70 (L70D) of the experiment, corresponding to 9–10 and 19–20 weeks old, respectively. Wilcoxon rank sum test was used to detect significant differences. *p < 0.05; **p < 0.01; ***p < 0.001.

The functional analysis result revealed a significant decrease in the abundance of aerobic bacteria in the Proteobacteria and Verrucomicrobia phyla, an increase in anaerobic bacteria in the Firmicutes phylum, and a decrease in the abundance of bacteria in the Bacteroidota phylum; however, there was no significant difference in the total abundance of anaerobic bacteria. Moreover, L70D rats exhibited a decrease in the abundance of facultative anaerobic bacteria in the Firmicutes phylum, alongside a reduction in potential pathogenic and stress-tolerant bacteria in the Bacteroidota phylum (Figure 11).

## 4 Discussion

Several studies have explored the impact of altitude on the abundance and diversity of the gut microbiota in humans and other mammals (Cui et al., 2023; Zhao et al., 2023; Zhang et al., 2023b). Altitude change has emerged as a major driving factor of fluctuations in the gut microbiota, influencing both bacterial composition and function. Different altitudes have been found to induce varying changes in the composition and abundance of the gut microbiota (Zhang et al., 2023a). A prior study investigating the characteristics of the intestinal microbiota of six geographical populations of *Macaca mulatta* in China found that *M. mulatta* populations in Tibet exhibited the highest intestinal microbiota diversity and possessed the highest number of unique microbiota (Zhao et al., 2018). Consistent with these findings, our research revealed an increase in intestinal microbiota diversity when rats were relocated to a high-altitude plateau environment. In this study, we characterized the dynamics of gut microbial changes in rats during high-altitude hypoxia and low-altitude reoxygenation. A significant increase in gut microbiota abundance and composition was observed on the 14th day of HAC, followed by a gradual return to lower abundances and compositions. Similar trends were observed during HADA; however, the specific microbiota types differed. Overall, this study aimed to provide insights into the alterations in intestinal microecology during HAC and HADA in natural environments. A comprehensive understanding of the gut microbiome characteristics during HAC and HADA will facilitate the effective development of gut microbiology–based intervention strategies for the prevention and treatment of altitude-related diseases.

In the current study, we determined that both HAC and HADA lead to a significant decrease in the relative abundance of *Lactobacillus* spp., reaching a minimum abundance at day 14, followed by a gradual increase. *Lactobacillus* spp. exhibit a diverse impact on host metabolic functions, with certain species being identified as probiotics that play a synergistic role with other genera to improve symptoms related to type 2 diabetes (Gurung et al., 2020). *Lactobacillus plantarum* strains have been shown to enhance the immune response of chickens in high-altitude regions (Wang et al., 2019). Conversely, in the present study, the observed reduction in the relative abundance of *Lactobacillus* during HAC and HADA may have contributed to the diminished stability of the rat gut microbiota.

The abundance of *Bacteroides* spp. has been found to significantly increase with altitude (Zhang et al., 2018b); species within this genus produce chain-breaking fatty acids that protect the intestinal tract, improve intestinal homeostasis, and enhance environmental adaptation. This increase in the abundance of *Bacteroides* has been associated with fat accumulation and increased energy storage (Ryan et al., 2014); additionally, these *Bacteroides* spp. enhance adaptation to the high-altitude of a plateau environment. Moreover, it is important to note that hypoxia is a key factor in inflammation; meanwhile, *Bacteroides* has been linked to the host’s inflammatory response (Lobionda et al., 2019). Therefore, *Bacteroides* likely plays an important role in both HAC and HADA.

In the current study, a rapid increase in the relative abundance of *Blautia* spp. was observed on the second day of HAC, followed by a sharp decrease to the pre-HAC level. *Blautia* spp. are strictly anaerobic bacteria capable of producing acetate, lactic acid, and succinate; these bacteria have been linked to conditions such as obesity and cardiovascular diseases (Shin et al., 2018; Zhu et al., 2018). In the present study, the *Blautia* genus was determined to be highly sensitive to changes in the plateau environment, suggesting a potential role in enhancing host adaptation to altitude changes. However, the precise mechanism underlying the interaction between *Blautia* and hypoxia remains unclear.

Our findings also suggest that fluctuations in oxygen concentrations in the environment may contribute to changes in the relative abundance of *Prevotella*. However, conflicting results regarding the abundance of *Prevotella* have been reported in a prior study; notably, this study identified *Prevotella* as the most abundant genus in the intestinal microbiota within a hyperbaric chamber (Han et al., 2022). In the current study, the absence of a control experimental design hindered our ability to distinguish the impact of oxygen from those of age. Age is an important factor that affects rat microbiota (Zhang et al., 2021). Prior research into the intestinal microbiota composition of rats throughout their lifespan revealed that the intestinal microbial diversity of young rats (Week 1–3) was higher than that of adult rats (Week 7–56), while the microbial diversity of old rats (Week 111) was observed to be the lowest (Chen et al., 2018). In the present study, the gut microbial alpha diversity of the LA group was found to significantly decrease throughout the 10 weeks of experimental analysis; meanwhile, the abundance of Firmicutes spp. significantly increased. This change in *Prevotella* abundance can be attributed to stress induced by changes in environmental oxygen concentration (Zeng et al., 2020). *Prevotella*, anaerobic bacteria associated with SCFA production, can effectively utilize carbohydrates to provide energy to the host via fermentation (Den Besten et al., 2013; White et al., 2014). Therefore, *Prevotella* spp. may mediate the microbiota response to HAC and HADA in rats. Moreover, *Prevotella* has been identified as the most enriched genus in wild house mice (*Mus musculus domesticus*) in Ecuador and Bolivia (nearly 4,000 m above sea level) (Suzuki et al., 2019). Thus, we hypothesize that *Prevotella* may be more abundant in species native to high altitude regions, but significantly less prevalent in species acclimatizing to these high altitudes. Prior studies support this understanding, demonstrating that the hypoxic environment of plateaus or simulated high altitudes can lead to a significant increase in the abundance of *Bacteroides* spp. alongside a concurrent decrease in *Prevotella* spp. in rat feces (Sun et al., 2020; Wang et al., 2023b).

The intestinal microbiota play a crucial role in energy and carbohydrate metabolism during short-term hypoxia (6–44 h); however, exposure to long-term hypoxia (4 weeks) has been found to suppress gut microbiota function (Lin et al., 2022). Our results align with this understanding, demonstrating that the abundance of bacterial populations associated with energy and carbohydrate metabolism significantly increased during the first 2 weeks of HAC, before exhibiting a significant decrease during the subsequent 2 weeks. During HADA, we observe an increase in the abundance of microbiota associated with energy metabolism, whereas the abundance of microbiota linked to carbohydrate metabolism peaked in the initial 4 days but declined thereafter. Oxygen levels may directly influence oxygen niches in anaerobic and aerobic bacteria. Under anoxic conditions, anaerobic bacteria may gain a competitive advantage over obligate anaerobic bacteria (Suzuki et al., 2019). Hypoxia induces a reduction in the abundance of total aerobic bacteria, an increase in total anaerobes and *Escherichia coli*, and an elevation in strict anaerobes, such as *Bifidobacterium*, *Anaplasma*, and *Lactobacillus*, alongside specific anaerobic groups, such as *Clostridium perfringens* and *Streptococcus gastricus* (Adak et al., 2014). High-altitude habituated populations have been found to possess significantly lower total aerobic bacteria levels and significantly higher abundances of obligate and facultative anaerobic bacteria (Adak et al., 2013). Our results further suggest that high-altitude, low-oxygen environments induce an expansion in the population of facultative anaerobes, which was also beneficial for the growth of obligate anaerobic bacteria. In response to the environmental selection pressure of high altitudes, the abundance of facultative anaerobes within the Proteobacteria phylum increased; then, during HADA, the abundance of aerobic bacteria in this phylum increased. However, the substantial reduction in aerobic and parthenogenetic anaerobic bacteria coupled with the significant increase in anaerobic bacteria within the Firmicutes phylum did not show signs of recovery even after 28 days following the elimination of high-altitude–associated environmental stress.

In the current study, we also established that exposure to high altitudes significantly reduces the body weight of rats, with this difference in body weight progressively increasing over 4 weeks. These findings are consistent with previous reports; specifically, these studies suggest that weight loss at high altitudes may be attributed to an elevated metabolic rate, reduced food intake, and alterations in the intestinal microbiota caused by high-altitude environments, which may induce gastrointestinal malabsorption and subsequent weight loss in rats (Lippl et al., 2010; Karl et al., 2018; Dünnwald et al., 2019). Furthermore, hypoxic exposure suppresses appetite (Bailey et al., 2015), which can likely be attributed to changes in the gut microbiota induced by low oxygen levels, resulting in weakened gastrointestinal digestion and absorption, further contributing to weight loss. However, in the present study, an altitude of 3,650 m above sea level did not significantly affect the daily activity levels of rats. Additionally, when the rats were returned to low altitudes, the difference in body weight between the control and experimental group gradually decreased over 4 weeks; however, the daily activity level of HADA rats was found to be significantly lower than that of LA rats. Upon returning to the plains (low-altitude) from the plateau (high-altitude), the rats must adapt to the increase in oxygen supply within the environment; this adjustment manifests as symptoms such as fatigue and drowsiness (He et al., 2019). It must be noted that HADA represents a reverse regulatory process that is closely linked to HAC.

This investigation reveals that during the process of high-altitude acclimatization and de-acclimatization, environmental changes trigger intricate ecological evolutions within the intestine. The patterns of gut microbiota changes exhibit species specificity. Some species can respond rapidly to environmental changes, while others may only begin to show changes in their species relative abundance after a certain period. This transformation in microbial community structure further affects the interactions among bacterial species and dynamic changes in community structure. These changes in gut microbiota are of great significance for the host to better adapt to the changes in altitude environment. Plateau pika exhibit higher microbial diversity and cellulase activity compared to low-altitude pika (Li et al., 2018). This discovery further emphasizes the crucial role of altitude changes in shaping the gut microbiota structure.

Our research has limitations, and we cannot exclude the impact of transportation stress on the gut microbiota. During transportation, various functions and systems of the organism are exposed to stress challenges, and the stability of the gut microbiota may also be affected. Existing studies have clearly shown that after experiencing transportation stress, adapting to new feeding environments, and changes in diet and management methods, cows exhibit the most significant differences in gut microbiota abundance on the second day (Maslen et al., 2022). Similarly, different shipment batches of mice can also have an impact on the gut microbiota, leading to particularly significant changes in low-abundance gut microbiota (Randall et al., 2019). These research results fully demonstrate that the changes in gut microbiota are influenced by a combination of multiple factors. During the data analysis process, it is crucial to choose appropriate statistical methods, which can help us more accurately identify the impact of key research variables on the gut microbiota. Moreover, combining previous research results for comprehensive analysis can help assess the universality and reliability of the obtained results. Overall, although the transportation process may be a covariate in this study, we believe that oxygen partial pressure is a key factor affecting changes in the gut microbiota. By deeply analyzing the intrinsic relationship between oxygen partial pressure and gut microbiota changes, and combining other possible influencing factors, we will strive to obtain more comprehensive research conclusions. This will help us to better understand the mechanism of gut microbiota changes under stress conditions and provide valuable references for future research.

In conclusion, our study identified a gradual increase and subsequent decrease in alpha diversity during acclimatization and de-acclimatization to high altitude. Prior studies on this subject have predominantly relied on simulated high-altitude hypoxia experiments (Zhang et al., 2018a). By contrast, our study specifically aimed to explore changes in the intestinal microbiota of rats during acclimatization and de-acclimatization to a natural high-altitude plateau environment. The inconsistency between our findings and those of previous studies may be attributed to the differences between a natural and simulated high-altitude hypoxic environment. Overall, this study has the potential to enhance the overall understanding of the homeostatic changes in the gut microbiota induced by HAC and HADA. These findings suggest that avoiding prolonged periods of ascent or descent could be advantageous in reducing the incidence of intestinal microbiological disorders.

## Data availability statement

Raw reads from amplicon sequencing were deposited in the NCBI Sequence Read Archive database (BioProject numbers: PRJNA910368, PRJNA909981, and PRJNA740353).

## Ethics statement

The animal study was approved by The Ethics Committee of Hospital of Chengdu Office of People’s Government of Tibetan Autonomous Region, China. The study was conducted in accordance with the local legislation and institutional requirements.

## Author contributions

DH: Data curation, Investigation, Methodology, Writing – original draft, Writing – review & editing. HN: Investigation, Methodology, Writing – review & editing. QZ: Visualization, Writing – review & editing. JS: Methodology, Writing – review & editing. CA: Visualization, Writing – review & editing. SW: Data curation, Writing – review & editing. CZ: Data curation, Writing – review & editing. SC: Data curation, Writing – review & editing. YF: Methodology, Writing – review & editing. YZ: Funding acquisition, Project administration, Resources, Writing – review & editing. ZH: Funding acquisition, Investigation, Project administration, Resources, Writing – review & editing.

## Footnotes

^1^https://docs.qiime2.org/2019.1/
