# Supplementary material for: Impact of high-altitude acclimatization and de-acclimatization on the intestinal microbiota of rats in a natural high-altitude environment
Source: Front Microbiol. 2024 May 7;15:1371247. doi: 10.3389/fmicb.2024.1371247 (PMC11106481; doi:10.3389/fmicb.2024.1371247)
Supplement: Supplementary file 1 [file Table_1.docx]

Supplementary Material

## Supplementary Figures


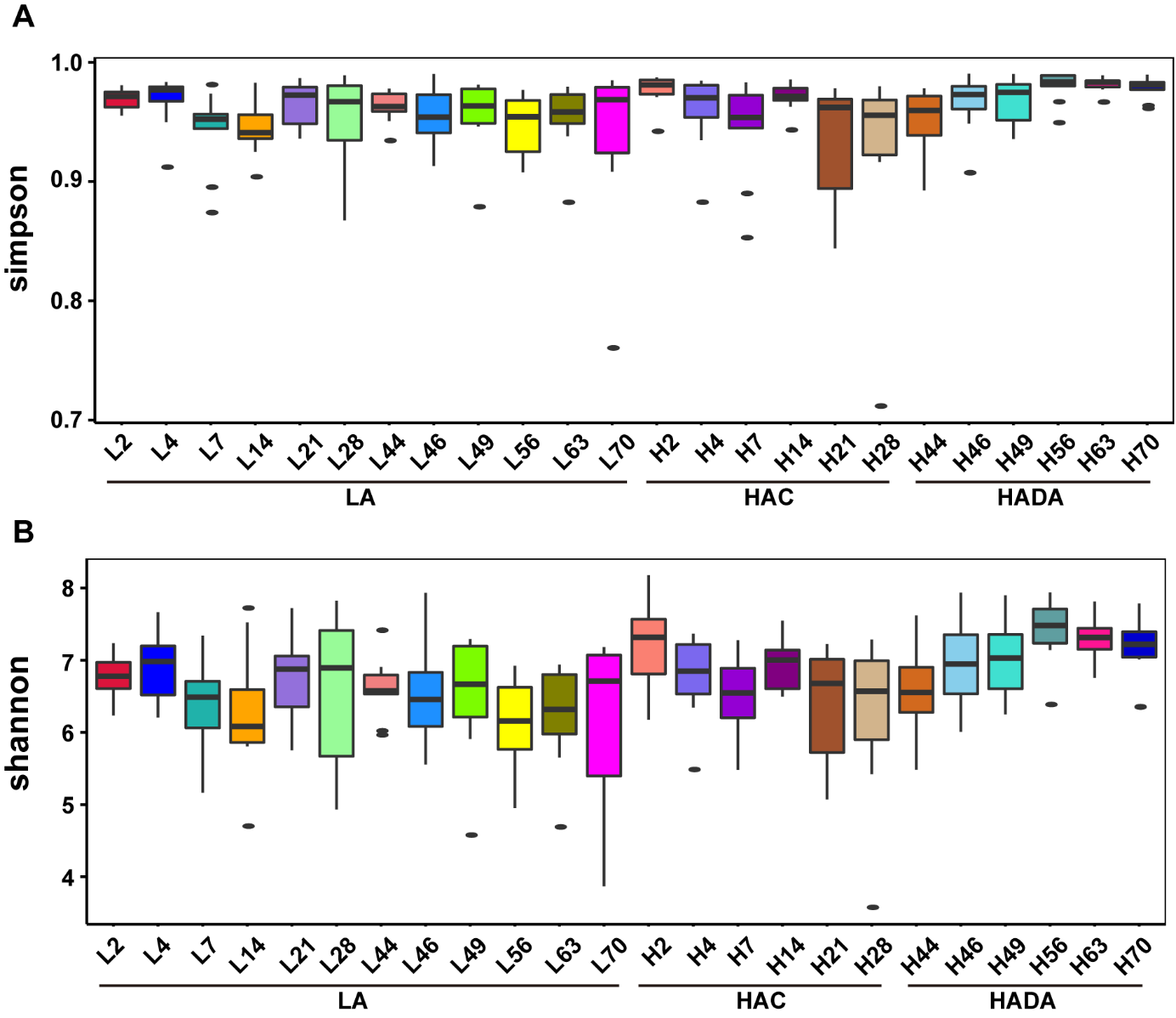


**Supplementary Figure 1.** **A** Alpha diversity was evaluated using the simpson index. **B** Alpha diversity was evaluated using the shannon index.
